# Comparison of Alemtuzumab and Anti-thymocyte Globulin Treatment for Acute Kidney Allograft Rejection

**DOI:** 10.3389/fimmu.2020.01332

**Published:** 2020-07-03

**Authors:** Marieke van der Zwan, Marian C. Clahsen-Van Groningen, Martijn W. F. van den Hoogen, Marcia M. L. Kho, Joke I. Roodnat, Katya A. L. Mauff, Dave L. Roelen, Madelon van Agteren, Carla C. Baan, Dennis A. Hesselink

**Affiliations:** ^1^Division of Nephrology and Transplantation, Department of Internal Medicine, Erasmus MC, University Medical Center, Rotterdam, Netherlands; ^2^Rotterdam Transplant Group, Erasmus MC, University Medical Center, Rotterdam, Netherlands; ^3^Department of Pathology, Erasmus MC, University Medical Center, Rotterdam, Netherlands; ^4^Department of Biostatistics, Erasmus MC, University Medical Center, Rotterdam, Netherlands; ^5^Department of Immunohematology and Blood Transfusion, Leiden University Medical Center, Leiden, Netherlands

**Keywords:** alemtuzumab, allograft rejection, rabbit anti-thymocyte globulin, kidney transplantation, T cell depletion

## Abstract

Rabbit anti-thymocyte globulin (rATG) is currently the treatment of choice for glucocorticoid-resistant, recurrent, or severe acute allograft rejection (AR). However, rATG is associated with severe infusion-related side effects. Alemtuzumab is incidentally given to kidney transplant recipients as treatment for AR. In the current study, the outcomes of patients treated with alemtuzumab for AR were compared with that of patients treated with rATG for AR. The patient-, allograft-, and infection-free survival and adverse events of 116 alemtuzumab-treated patients were compared with those of 108 patients treated with rATG for AR. Propensity scores were used to control for differences between the two groups. Patient- and allograft survival of patients treated with either alemtuzumab or rATG were not different [hazard ratio (HR) 1.14, 95%-confidence interval (CI) 0.48–2.69, *p* = 0.77, and HR 0.82, 95%-CI 0.45–1.5, *p* = 0.52, respectively). Infection-free survival after alemtuzumab treatment was superior compared with that of rATG-treated patients (HR 0.41, 95%-CI 0.25–0.68, *p* < 0.002). Infusion-related adverse events occurred less frequently after alemtuzumab treatment. Alemtuzumab therapy may therefore be an alternative therapy for glucocorticoid-resistant, recurrent, or severe acute kidney transplant rejection.

## Introduction

Alemtuzumab is incidentally used to treat acute kidney allograft rejection (AR) (1–5). Alemtuzumab is a humanized monoclonal rat antibody directed against the cell surface glycoprotein CD52 (6). Treatment with alemtuzumab causes a long-lasting depletion of various cells of the adaptive (T- and B cells) and innate immune system (NK cells, dendritic cells, monocytes, and granulocytes) (6). The drug is registered for the treatment of relapsing-remitting multiple sclerosis (7). The Campath® Distribution Program offers off-label treatment with alemtuzumab for other indications, including therapy for kidney transplant recipients and patients with chronic lymphocytic leukemia (8).

Currently, rabbit anti-thymocyte globulin (rATG) is the treatment of choice for glucocorticoid-resistant, recurrent or severe (Banff grade IIA or higher) acute T cell-mediated rejection (aTCMR) (9). Although effective, rATG has several limitations, for instance infusion-related side effects (10–12). Alemtuzumab might be an alternative T cell-depleting therapy for AR with fewer infusion-related side effects (1–5).

The outcomes of alemtuzumab therapy for AR in kidney transplant recipients have only been reported in five small case series (with a cumulative number of 88 patients), concluding that patients with AR responded well to therapy with alemtuzumab (1–5). However, in only one of these reports, alemtuzumab was compared to rATG therapy and none of them were randomized controlled trials (1). Our center participated in one of these case series (1). In this case series, 11 patients with AR and a contra-indication for rATG were treated with alemtuzumab. The incidence of the composite endpoint “treatment failure” was comparable between both groups (alemtuzumab 27% vs. rATG 40%, *p* = 0.89) and treatment with alemtuzumab was associated with fewer infusion related side effects and reduced costs (1).

Since 2012 and after our initial positive experience with alemtuzumab, it became the treatment of choice for all patients with glucocorticoid-resistant, severe or recurrent AR in the Erasmus MC (1). Here, we present further data on patient- and allograft outcome on subsequent patients treated with alemtuzumab for AR in our center. Factors that influenced allograft survival were investigated, and we focused on the occurrence of infections, malignancies and autoimmune diseases. Patient-, allograft-, and infection-free survival of alemtuzumab-treated patients were compared with those of patients treated with rATG for AR (10).

## Materials and Methods

### Study Design

A retrospective analysis was performed on data of kidney transplant recipients who were treated in the Erasmus MC, University Medical Center Rotterdam, with alemtuzumab (Campath®, Sanofi Genzyme, United States) because of AR between January 2012 and January 2018. The study was approved by the medical ethical review board of the Erasmus MC (number 2018-1430). The patients were identified by the electronic medication prescription system of our hospital pharmacy. Patients with blood group AB0-incompatible kidney transplantations were excluded from the analysis, because they receive alemtuzumab as induction therapy (13).

The outcomes were compared to those of a cohort of patients treated with rATG (Thymoglobulin®, Sanofi Genzyme, United States) for AR between January 2002 and January 2012. The characteristics and outcomes of this cohort were described in detail previously (10).

All AR episodes (including recurrent AR) were biopsy-proven and biopsies were re-evaluated according to the Banff 2015 (for rATG-treated patients) and Banff 2017 classification (for alemtuzumab-treated patients) by one dedicated renal-pathologist (M.C.C-v.G.) (14–16). The presence of donor-specific anti-HLA antibodies (DSA) and non-donor-specific HLA antibodies against HLA-A, HLA-B, HLA-DR, and HLA-DQ were examined in alemtuzumab-treated patients using the single-antigen bead Luminex assay on serum samples collected at the time of AR. DSA directed against Cw and DP HLA molecules were not tested. The presence of DSA was not routinely tested in the period 2002–2012 when rATG still was the therapy of choice (10). Therefore, the biopsies of the rATG-treated patients could not be reclassified according to the Banff 2017 criteria (14).

Of patients treated with alemtuzumab, patient survival, allograft function [estimated glomerular filtration rate (eGFR); Chronic Kidney Disease Epidemiology Collaboration (CKD-EPI) (17)], allograft survival (censored for death), variables that could influence allograft survival (patient and donor characteristics, type of immunosuppressive therapy, and type and grade of rejection), and adverse events were assessed. Baseline eGFR was defined as the highest eGFR in the 3 months prior to AR. Delayed graft function (DGF) was defined as the need for dialysis in the first week after transplantation. Allograft loss was defined as the need for dialysis or retransplantation. The follow-up period for allograft loss and infection was from the day of T cell-depleting therapy until death, retransplantation, or loss to follow-up. Malignancies and mortality were evaluated until the last follow-up visit, which could be after subsequent kidney transplantation. The Dutch national pathology archive PALGA (Pathologisch-Anatomisch Landelijk Geautomatiseerd Archief, https://www.palga.nl/) was used for collecting of data relating to the occurrence of malignancy. Infections were considered serious if the infection necessitated hospitalization or occurred during hospital admission for another reason.

The allograft- and patient survival data of patients who had received a kidney transplant in the same time periods in our center and were not treated with T cell-depleting therapy was also compared to the patients treated with T cell-depleting therapy for AR.

### Maintenance Immunosuppressive Therapy

The standard immunosuppressive regimen included induction therapy with basiliximab (Simulect®, Novartis Pharma, Basel, Switzerland) 20 mg intravenously on days 0 and 4 after transplantation, followed by maintenance therapy with tacrolimus (Prograf®, Astellas Pharma, Leiden, the Netherlands), mycophenolate mofetil (MMF; Cellcept®, Roche Pharmaceuticals, Basel, Switzerland) and glucocorticoids.

Basiliximab became part of our standard immunosuppressive regimen in 2009. Dosing of tacrolimus and MMF was based on pre-dose concentrations (C_0_). Target C_0_ for tacrolimus was, respectively, 10–15 μg/L (weeks 1–2), 8–12 μg/L (weeks 3–4), 5–10 μg/L (weeks 5–12), and 4–8 μg/L from month 4 onwards. MMF was started at 1,000 mg twice daily and subsequent dosing was based on C_0_ (target C_0_ was 1.5–3.0 mg/L). Glucocorticoids were given as an intravenous dose of 100 mg on days 0–3 and followed by a dose of 20 mg/day (days 4–14). Thereafter, glucocorticoids were tapered off and completely withdrawn around month 4.

### Treatment of AR

The first-line treatment of aTCMR was methylprednisolone 1,000 mg (Solu-Medrol®, Pfizer, New York, the United States) intravenously daily for 3 consecutive days, followed by a second-line treatment with alemtuzumab or rATG in case of a glucocorticoid-resistant, recurrent or severe aTCMR (Banff grade IIA or higher). rATG was administered as a single bolus [4 mg/kg (actual bodyweight, no maximum dose limit)] intravenously (10). Alemtuzumab was administered subcutaneously (18). The first 14 patients were treated with alemtuzumab (30 mg) daily for 2 consecutive days. Since T cell-depletion already occurred after one dose of alemtuzumab, the next patients received a single dose (30 mg). To prevent infusion-related side effects patients were premedicated with glucocorticoids (50 mg intravenously), acetaminophen (4 times daily 1,000 mg), and clemastine (4 mg intravenously). The alemtuzumab-treated patients were discharged the same day if no severe side-effects were noted. T- and B cell counts were measured with BD FACSCanto™ software every 3 months until the T cell count was >200 × 10^6^/L. In the patients treated with rATG, a CD3^+^ T cell count <200 × 10^6^/L was aimed for a duration of 2 weeks during which patients were hospitalized (10). If CD3^+^ T cell counts increased during this period, a repeat dose of rATG was administered. All patients received prophylaxis for *Pneumocystis jirovecii* (sulfamethoxazole/trimethoprim) and cytomegalovirus (CMV; valganciclovir, except for CMV seronegative recipients with CMV seronegative donors) until the T cell count was >200 × 10^6^/L. Patients with aABMR or mixed type AR could additionally be treated with intravenous immunoglobulins (IVIg), plasma-exchange, or both according to the Kidney Disease: Improving Global Outcomes (KDIGO) guideline (9).

### Statistical Methods

Categorical variables are presented as number (percentage). Continuous variables are presented as mean with standard deviation for normally distributed variables or median with interquartile range (IQR) for non-normally distributed variables. For differences between unpaired non-normally distributed continuous data or unpaired categorical data, the Kruskal-Wallis and Mann-Whitney *U*-tests, and the Chi-squared and Fisher's exact tests were used. Kaplan-Meier survival analysis was used to examine subgroups (e.g., age categories and rejection types) within the alemtuzumab group and to compare allograft- and patient survival between alemtuzumab-treated patients and patients transplanted in the same period and who were not treated with alemtuzumab.

The influence of predictor variables on allograft survival in alemtuzumab-treated patients was analyzed with multivariable Cox proportional hazard regression analysis. Due to the number of events (41 allograft losses), the number of variables that could be included per analysis was limited. The influence of the most significant variable was tested in the presence of all the other variables one by one, and the non-significant variables were eliminated from the model by backward elimination.

Propensity scores were used to control for baseline differences between the patients treated with rATG and alemtuzumab (19). They were acquired by performing a logistic regression with therapy type as the outcome variable. Covariates included in the logistic model were: age of the patient at time of AR, gender, primary kidney disease, donor type (living/deceased), induction therapy (43% of patients treated with rATG received induction therapy, vs. 97.3% of alemtuzumab treated patients), maintenance therapy, time to AR, and type of AR. The resulting propensity score was used as a covariate in Cox proportional hazards regression models (for calculation of the patient-, allograft-, and infection-free survival), in linear regression models (for continuous outcomes), and in logistic regression models (for categorical outcomes).

A two-sided *p* < 0.05 was considered statistically significant. GraphPad Prism, version 5 (San Diego, CA, USA), SPSS version 21 (SPSS Inc., Chicago, IL, USA), and R (R Foundation for Statistical Computing, Vienna, Austria, version 3.5.1) were used for the statistical analysis.

## Results

### Patient Demographics

Between January 2012 and January 2018, 1,214 patients received a kidney transplant at our center. Of these, 113 patients (9.3%) were treated with alemtuzumab for AR. Three patients were treated with alemtuzumab twice because of two separate rejection episodes of the same kidney transplant. Between January 2002 and January 2012, 1,107 patients were transplanted with a kidney and 108 patients of these (9.8%) were treated with rATG for AR (10). The median cumulative dose of rATG per patient was 7.4 mg/kg. Baseline characteristics of patients treated with either alemtuzumab or rATG for AR are presented in Table 1.

**Table 1:** Baseline characteristics of patients treated with either alemtuzumab or rATG.

**Characteristic**	**Alemtuzumab (*n* = 113)**	**rATG (n=108)**	***p*-value**
Recipient age at transplantation—yr.	56 (39–63)	45 (34–55)	0.0001
Recipient age at rejection—yr.	56 (40–63)	46 (35–56)	0.0002
Donor age—yr.	54 (43–63)	54 (46–61)	0.82
Female sex-no. (%)	47 (40.5)	40 (37.0)	0.69
Cause of ESRD-no.			
DM/HTN/GN/PKD/	26/22/21/9/7/27/4	23/10/18/16/17/16/3	0.04
reflux/other^*^/unknown			
Ethnic distribution-no.			
Caucasian/Black/	79/17/8/11/1	70/16/5/5/7	0.12
Asian/Arab/other			
Transplant number-no.			
1/2/3	88/22/6	76/25/5	0.71
Preemptive kidney transplantation-no. (%)	41 (35.3)	25 (23.4)	0.06
Donor type-no.			
LR/LUR/DBD/DCD	27/55/12/22	35/47/15/10	0.12
HLA mismatch			
HLA A: 0/1/2	26/61/29	21/60/23	0.75
HLA B: 0/1/2	10/53/53	11/55/38	0.39
HLA DR: 0/1/2	21/55/40	13/50/41	0.48
PRA actual-no. (%)			0.52
0–5%	93 (80.2)	81 (77.1)	
6–83%	22 (19.0)	21 (20)	
84–100%	1 (0.8)	3 (2.9)	
PRA peak-no. (%)			0.15
0–5%	69 (59.5)	62 (59)	
6–83%	31 (26.7)	32 (30.5)	
84–100%	16 (13.8)	11 (9.5)	
CMV IgG serostatus recipient-no. (%)			
Positive	83 (73.6)	75 (70.8)	0.76
EBV IgG serostatus recipient-no. (%)			
Positive	106 (93.8)	90 (92.8)	0.78

*Data are numbers (%) or median (interquartile range)*.

* *Other kidney diseases included focal segmental glomerulosclerosis, vascular disease, septic shock, kidney dysplasia/nephrectomy, congenital nephrotic syndrome, Alport syndrome, nephronophtisis, drug intoxication, RCAD syndrome, or tubulointerstitial nephritis. Data of rATG-treated patients are prescribed previously (10). CMV, cytomegalovirus; DBD, donation after brain death; DCD, donation after circulatory death; DM, diabetes mellitus; EBV, Epstein-Barr virus; ESRD, end stage renal disease; GN, glomerulonephritis; HLA, human leucocyte antigen; HTN, hypertensive nephropathy; LR, living related; LUR, living unrelated; PKD, polycystic kidney disease; PRA, panel reactive antibody; rATG, rabbit anti-thymocyte globulin*.

Induction therapy with basiliximab was given to 303 (27.4%) of all patients transplanted between 2002 and 2012 (the rATG period) and to 1,065 (87.8%) of all patients between 2012 and 2018 (the alemtuzumab period). As a result, significantly more patients treated with alemtuzumab (93.8%) had previously received basiliximab induction therapy compared to rATG-treated patients [29.2%; *p* < 0.0001 (Table 2)]. A tacrolimus- and MMF-based maintenance therapy was given to 81% of alemtuzumab-treated patients and to 72.2% of rATG-treated patients (*p* = 0.08; Table 2). First line therapy for AR was methylprednisolone in 94.8% of alemtuzumab-treated patients, and to 86.1% or rATG-treated patients (Table 2).

**Table 2:** Immunosuppressive therapy in patients treated with alemtuzumab or rATG.

**Immunosuppressive therapy**	**Alemtuzumab (*n* = 113)**	**rATG (*n* = 108)**	***p*-value**
Induction therapy-no. (%)			<0.0001
None	3 (2.7)^*^	62 (57.4)	
Basiliximab	106 (93.8)	33 (29.2)	
rATG	2 (1.8)	10 (8.8)	
Rituximab	2 (1.8)	0 (0)	
Daclizumab	0 (0)	2 (1.9)	
Maintenance immunosuppression- no. (%)			0.08
TAC/MMF/glucocorticoids	78 (67.2)	58 (53.7)	
TAC/MMF	16 (13.8)	20 (18.5)	
TAC + other (non-MMF)	11 (9.5)	6 (5.6)	
MMF + other (non-TAC)	11 (9.5)	20 (18.5)	
Anti-rejection therapy-no. (%)			
Methylprednisolone prior to T cell-depleting therapy	110 (94.8)	93 (86.1)	0.04
Cumulative dose of methylprednisolone, mg			0.004
1,000	0 (0)	2 (2.2)	
2,000	1 (0.9)	9 (8.2)	
3,000	96 (87.3)	79 (71.8)	
4,000	1 (0.9)	0 (0)	
6,000	12 (10.9)	3 (2.7)	
Additional anti-rejection therapy in patients with ABMR			
Intravenous immunoglobulins	10	1	
Plasma-exchange + intravenous immunoglobulins	3	2	
Additional anti-rejection therapy in patients with mixed AR			
Intravenous immunoglobulins	8	4	
Plasma-exchange + intravenous immunoglobulins	0	4	

*Data are numbers (%)*.

* *In three patients no induction therapy was administered because of an HLA-identical donor. TAC + other regime contained combinations of TAC, glucocorticoids, everolimus, or azathioprine. Other combinations existed of a combination of azathioprine, glucocorticoids, everolimus, cyclosporine A, AEB071, or FTY720. MMF + other regime contained combinations of MMF, glucocorticoids, cyclosporine A, everolimus, or belatacept. Data of rATG-treated patients are prescribed previously (10). MMF, mycophenolate mofetil; TAC, tacrolimus*.

Sixty-four alemtuzumab-treated patients (55.2%) and 64 (59.3%) rATG-treated patients had an early AR (within 3 months after transplantation; Table 3). The distribution of the Banff grade of AR was not different between the patients treated with alemtuzumab or rATG (*p* = 0.89; Table 3). In 18 patients (15.5%), a second kidney allograft biopsy was performed after the initial treatment with methylprednisolone and immediately before alemtuzumab treatment to confirm ongoing AR (Table S1).

**Table 3:** Rejection characteristics.

**Rejection characteristic**	**Alemtuzumab (*n* = 116)^$^**	**rATG (*n* = 108)**	***p*-value**
Time to rejection—days	32 (2–1644)	24 (8–339)	0.83
Early rejection (<3 months)-no. (%)	64 (55.2)	64 (59.3)	0.59
Late rejection (>3 months)-no. (%)	52 (44.8)	44 (40.7)	0.59
Delayed graft function during AR	33 (28)	19 (17.6)	0.06
Banff classification-no.^*^			0.89
aTCMR			
aTCMR IA/IB	1/9	6/8	
aTCMR IIA/IIB/III	29/23/2	21/20/1	
Borderline aTCMR	3	0	
ABMR			
aABMR	17	12^@^	
DSA+ and C4d+	7		
DSA+ and C4d-	0		
DSA- and C4d+	7		
C4d+, no DSA tested	2		
Histologic features of ABMR, no DSA/C4d	1^¥^		
c/aABMR	1^§^	3	
Mixed aTCMR with aABMR			
aTCMR I/II/III	9/7/2	8/10/0^@^	
DSA+ and C4d+	5		
DSA+ and C4d-	6		
DSA- and C4d+	4		
C4d+, no DSA tested	3		
Mixed aTCMR with c/aABMR	1^$^	1	

*Data are numbers (%) or median (interquartile range)*.

$ A total of 113 patients were treated with alemtuzumab, however three patients were treated with alemtuzumab twice because of two separate rejection episodes of the same kidney transplant

* *Banff classification of aTCMR, ABMR and mixed AR were compared. Re-classification in 12 biopsies of alemtuzumab-treated patients and 18 biopsies of rATG-treated patients was not possible because the biopsies were missing from archives. The primary pathological diagnosis of these biopsies was aTCMR in five patients, ABMR in two patients, and mixed AR in five patients. Data of rATG-treated patients are prescribed previously (10)*.

¥ *Histologic features of ABMR with glomerulitis and peritubular capillaritis, but C4d staining was negative and no DSAs were present*.

§ *The patients with c/aABMR had no DSAs and C4d staining was positive in the peritubular capillaries*.

*^$^The patient with mixed c/aABMR had DSAs and C4d staining was negative*.

@ *DSAs were not routinely measured in the rATG-treated patients. ABMR antibody mediated rejection; aABMR, active antibody mediated rejection; aTCMR, acute T cell mediated rejection; c/aABMR, chronic/active antibody mediated rejection; DGF, Delayed graft function (need for dialysis in the first week after transplantation); DSA, de novo donor specific antibodies*.

### Patient Survival

**Figure 1:**
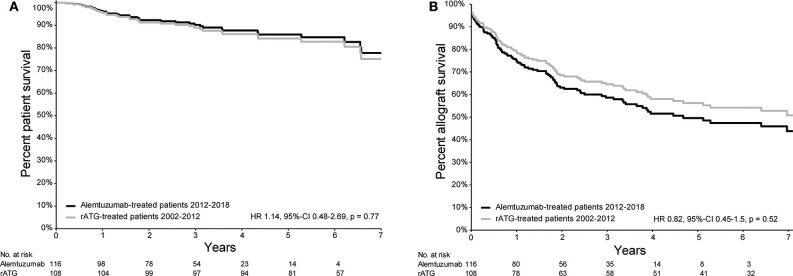
Survival plots of patient- and allograft survival of patients with acute rejection and treated with either rATG or alemtuzumab. **(A)** Patient survival curve (from the time of treatment) based on the propensity score Cox regression model of patients treated with either alemtuzumab (2012–2018) or rATG (2002–2012) for acute kidney allograft rejection. **(B)** Allograft survival curve (from the time of treatment) based on the propensity score Cox regression model (event = allograft loss, censored for death) of patients treated with either alemtuzumab (2012–2018) or rATG (2002–2012) for acute kidney allograft rejection.

**Figure 2:**
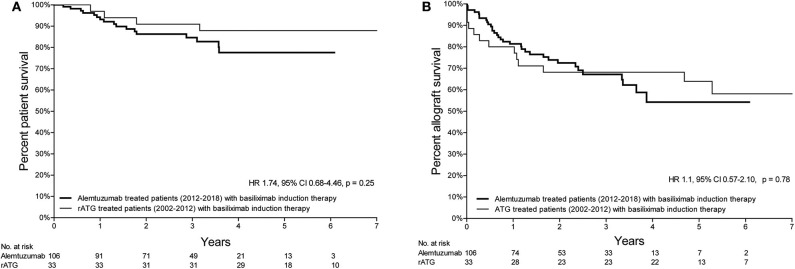
Kaplan-Meier survival curves of the patient- and allograft survival of patients treated with basiliximab induction therapy. **(A)** Kaplan-Meier curve of the patient survival curve (from the time of treatment) of patients treated with either alemtuzumab (2012–2018) or rATG (2002–2012) for AR and who received induction therapy with basiliximab. **(B)** Kaplan-Meier curve of the allograft survival curve (from the time of treatment) of patients treated with either alemtuzumab (2012–2018) or rATG (2002–2012) for AR and who received induction therapy with basiliximab.

Patient survival of patients treated with either alemtuzumab or rATG for AR is depicted in Figure 1A. Compared with the historical rATG cohort, the patient survival of the alemtuzumab group was not different [hazard ratio (HR) 1.14, 95%-confidence interval (CI) 0.48–2.69, *p* = 0.77; Figure 1A), also when only those patients who were treated with basiliximab induction therapy were included (HR 1.74, 95%-CI 0.68–4.46, *p* = 0.25; Figure 2A).

The patient survival of alemtuzumab-treated patients was significantly lower compared with the patients transplanted in the same period and who were not treated with alemtuzumab (HR 2.38, 95%-CI 1.25–4.54, *p* = 0.0036, Figure S1A). In the total follow-up period [median 2.8 years (IQR 1.3–3.8 years)], 18 patients died after a median of 1.45 years (IQR 0.92–2.93; Table S2). A univariable Cox proportional hazard regression analysis was performed to investigate which variables influenced the risk of death in patients treated with alemtuzumab. The only variable that was associated with the risk of death was age of the recipient (HR per year 1.09, 95%-CI 1.04–1.14, *p* < 0.0001; Table S3). This increased risk of death was seen in alemtuzumab-treated patients older than 50 years at the time of transplantation (Figure S2).

A comparison between the patient survival of rATG-treated and patients transplanted in the same period and who were not treated with rATG is shown in Figure S1C and was described previously (10).

### Kidney Allograft Survival

Death-censored kidney allograft survival of alemtuzumab-treated patients was not different compared to that of patients who received rATG for AR (HR 0.82, 95%-CI 0.45–1.50, *p* = 0.52; Figure 1B). A similar survival was also observed when only those patients who were treated with basiliximab induction therapy were included (HR 1.10, 95%-CI 0.57–2.10, *p* = 0.78; Figure 2B). Additional information about the kidney allograft function after alemtuzumab or rATG therapy for AR is provided in Figure S3.

The allograft survival of alemtuzumab-treated patients was significantly worse compared to the allograft survival of patients that were transplanted in the same period and who were not treated with alemtuzumab (HR 258.0, 95%-CI 112.0–591.3, *p* < 0.0001; Figure S1B). During the follow-up (median 2.2 years, IQR 1–3.5), 41 (35.3%) patients lost their kidney allograft after alemtuzumab therapy for AR, of which six never had a functioning graft [primary non-function (PNF)]. To investigate which variables influenced allograft survival in alemtuzumab-treated patients, a Cox proportional hazard regression analysis was performed. In the univariable model, age of the recipient, number of HLA mismatches, glucocorticoid maintenance treatment, timing of AR, and the Δ eGFR (percentage change between baseline eGFR and eGFR at the moment of AR) significantly influenced the risk for death-censored allograft loss (*p* < 0.05, Table S4) in alemtuzumab-treated patients. The variables glucocorticoid use and timing of rejection were related because all patients with an early acute rejection used glucocorticoids as maintenance therapy, while only 56.6% of patients with a late acute rejection used glucocorticoids (*p* < 0.0001). The Banff grade of rejection did not influence allograft survival (*p* = 0.19). Allograft survival of alemtuzumab-treated patients suffering from either aTCMR or aABMR is shown in Figure S4.

**Figure 3:**
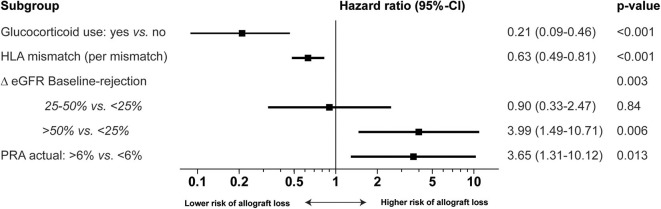
Multivariable Cox proportional hazard regression analysis of risk for allograft loss in alemtuzumab-treated patients. Multivariable analysis of the risk of allograft loss with hazard ratio [Exp(B), 95%-confidence interval and *p*-value]. Delta (Δ) eGFR baseline-moment of rejection is the percentage change between the baseline eGFR and eGFR at the moment of rejection. Glucocorticoid use means maintenance therapy with glucocorticoids during the rejection. PRA Panel reactive antibodies.

The final multivariable model showed that patients with actual panel reactive antibodies (PRA) >6%, and patients with a Δ eGFR of more than 50% had an inferior allograft survival after alemtuzumab therapy (Figure 3). Patients using glucocorticoids at time of AR, and patients with more HLA mismatches, showed a superior allograft survival after alemtuzumab therapy (Figure 3). Several variables were compared between patients with an HLA mismatch of 0–3 and an HLA mismatch of 4–6 (Table S5). One variable was significantly different: 42 (72%) recipients with 4–6 HLA mismatches received a living unrelated donor kidney (Table S5), while 13 (23%) recipients with 0–3 HLA received a living unrelated donor kidney (*p* < 0.001; Table S5).

The allograft survival of patients treated with survival of rATG was worse compared to that of patients who were not treated with rATG and transplanted in the same time period (HR 15.9, 95%-CI 9.2–27.4, *p* < 0.0001; Figure S1D). A multivariable Cox proportional hazard regression analysis was performed for patients treated with rATG for AR and reported previously (10). This analysis demonstrated that allograft survival was superior in patients with an early AR compared with a late AR (10).

### Adverse Events

Infusion-related side effects also occurred less frequently in alemtuzumab-treated patients compared with the patients treated with rATG (Table 4). No alemtuzumab-treated patients experienced serum sickness vs. five patients in the rATG group (*p* = 0.02). No patients experienced cytokine release syndrome or pulmonary edema after alemtuzumab. The median duration of hospitalization was 3 days (IQR 1–6) in patients treated with alemtuzumab and 15 days (IQR 13–19) in rATG-treated patients.

**Table 4:** Adverse events after therapy with alemtuzumab or rATG.

**Adverse events**	**Alemtuzumab**	**rATG**	***p*-value**
Fever^*^-no. (%)	10 (8%)	42 (61.8%)	<0.001
Systolic blood pressure <90 mmHg-no. (%)	1 (0.8%	7 (10.4%)	0.003
Tachycardia >100/min-no. (%)	18 (15.5%)	44 (69.8%)	<0.001
Interventions-no.			
Transfer to ICU	1 (0.9%)	5 (4.6%)	0.11
Supplemental oxygen	1 (0.9%)	9 (13.4%)	0.03
Volume resuscitation	0	6 (9.0%)	0.06

*Data are numbers (percentage) and median (interquartile range). Fever, blood pressure, tachycardia and interventions were registered in the 24 h after therapy. Under-reporting of the incidence of infusion-related adverse events is possible in 37 patients who were dismissed on the day of alemtuzumab therapy. Data of rATG-treated patients are prescribed previously (10)*.

* *Fever was defined as temperature above 38.5°C*.

**Figure 4:**
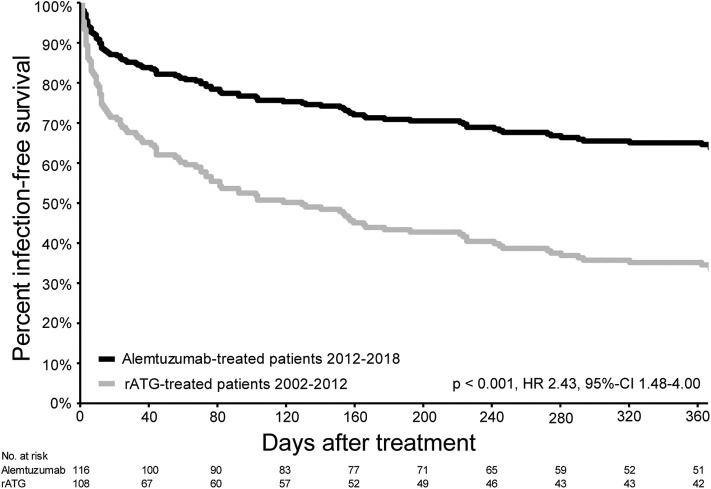
Infection-free survival in the first year after treatment for acute rejection. Infection-free survival (excluding CMV, EBV, and BK virus infections) of patients with AR and treated with alemtuzumab (2012–2018) and patients treated with rATG for AR between 2002 and 2012.

The infection-free survival (excluding CMV, EBV, and BK virus infections) in the first year after alemtuzumab treatment was significantly better compared with the infection-free survival of the rATG-treated patients (HR 0.41, 95%-CI 0.25–0.68, *p* < 0.002; Figure 4). CMV reactivation occurred in 25 patients (21.6%) treated with alemtuzumab (Table S6), compared to 27 patients (25%) in the rATG group (*p* = 0.10). In both the alemtuzumab- and rATG-treated groups, two patients experienced a primary CMV infection (*p* = 0.50). Additional information on the occurrence of infections is presented in Table S6.

Secondary autoimmune events have been described after administration of alemtuzumab (6). In the current study, two patients developed inflammatory polyneuropathy (one case of Guillain-Barre syndrome and one case of chronic inflammatory demyelinating polyneuropathy) after alemtuzumab treatment (20). No patients were diagnosed with autoimmune thyroid disorders, idiopathic thrombocytopenic purpura or autoimmune nephropathy.

Repopulation of T cells >200 × 10^6^/L occurred in 55.7% of alemtuzumab-treated patients in the first year after administration (Figures S5A,B). In 40.2% of the patients, repopulation of B cells >100 × 10^6^/L (Figures S5C,D) was seen at 1 year.

Solid tumors were diagnosed in seven alemtuzumab-treated patients during the total follow-up [median 2.8 years (IQR 1.3–3.8); Table S7]. The incidence of solid tumors was 2.3 per 100 person-years with a median time after alemtuzumab therapy of 28 months (IQR 9–38), and the age at the time of diagnosis was 65 years (IQR 60–76). Seven patients were diagnosed with skin cancer: 21 basal cell carcinomas and 12 squamous cell carcinomas. In the rATG treated patients, 14 malignancies were diagnosed after a mean time of 63 months (standard deviation 45 months) during the follow-up of 6.8 years (IQR 4.9–9.1; Table S8) (10).

## Discussion

In the current study, the largest cohort of patients treated with alemtuzumab for AR is described. The results of this study suggest that alemtuzumab could be an alternative to rATG for the treatment of glucocorticoid-resistant, severe or recurrent AR. Compared to rATG, allograft- and patient survival of patients treated with alemtuzumab was not different. Moreover, adverse events and infections seemed to occur less frequently in patients treated with alemtuzumab compared with rATG-treated patients.

Alemtuzumab seems as effective as rATG for the prevention of allograft loss after AR. Five case series have described allograft outcome in patients treated with alemtuzumab for AR (1–5). The results of these case series are difficult to compare with our study. Four of the five studies were performed more than 15 years ago and patients in these studies were not treated with the current gold standard therapy (induction therapy in combination with tacrolimus and MMF) (2–5). Furthermore, these case series were of heterogeneous design. First, alemtuzumab was prescribed as first line treatment for AR in two studies (4, 5), and in one study alemtuzumab was prescribed to patients with AR resistant to ATG or OKT3 (2). Second, the dose of alemtuzumab ranged from 15 mg (1) to 93 mg (2). Third, the follow-up period of these studies ranged from 3 months (1) to 10 years (4). Compared with treatment with methylprednisolone or rATG, allograft survival in alemtuzumab-treated patients was similar (1, 4). The result of our study supports this conclusion.

Treatment with alemtuzumab is associated with serious side effects and therefore the assessment of the benefit-risk balance in the individual patient before initiation of treatment is necessary. We investigated which clinical factors influenced allograft survival. Factors that were associated with a good response were a low Δ eGFR between baseline and the moment of AR, glucocorticoid maintenance therapy at the time or AR, and an actual PRA below 6%. The use of glucocorticoid maintenance therapy and timing of rejection were related, because all patients with an early acute rejection used glucocorticoids as maintenance therapy. Therefore, we are not sure if glucocorticoid use is a protective factor, or that an early rejection is associated with a better allograft outcome compared with a late rejection. Late rejections occur in patients who visit the outpatient clinic less frequently and with intervals of 1–4 months, likely leading to a delay in diagnosis.

Surprisingly, patients with more HLA mismatches had a better response to alemtuzumab therapy compared with those with less HLA mismatches. Analysis of all factors showed that patients with more HLA mismatches (4–6) more often received a kidney from a living unrelated donor. How this is related to a better response to alemtuzumab treatment is unclear. It is known that results of living donor kidney transplantation are better compared to deceased donor transplantation, even with higher numbers of HLA mismatches (21). Taken together, based on these results, we treat patients with an early AR aggressively with alemtuzumab and are more reluctant to administer alemtuzumab in patients with a late AR who also have a considerable loss of renal function.

Seventeen patients with aABMR were treated with alemtuzumab. Treatment options for aABMR are limited and no specific drugs have received US Food and Drug Administration approval. Currently, the therapy for aABMR consists of glucocorticoids, IVIg and/or plasma-exchange, although the evidence for this treatment is not strong (22, 23). Since alemtuzumab causes lysis of T- and B cells, as well as antigen presenting cells, alemtuzumab may be considered in patients with aABMR. In our study, patients with aABMR showed a good response to alemtuzumab therapy. However, larger studies are necessary to confirm our results and analyze the best therapeutic strategy.

Although T cell-depletion after alemtuzumab therapy lasts longer than after rATG (24), the infection-free survival was better after therapy with alemtuzumab compared with rATG. The biggest difference in the number of infections in patients treated with rATG or alemtuzumab occurred in the first few weeks after therapy (Table S6). A possible explanation for this is the longer duration of hospitalization after therapy for AR in rATG-treated patients compared with alemtuzumab-treated patients. A longer hospitalization is associated with a higher risk for health care-associated infections (25). The occurrence of CMV disease or reactivation was similar between patients treated with alemtuzumab or rATG. In literature, similar results (lower frequency of infections and no difference in CMV infections) are seen when alemtuzumab or rATG are used as induction therapy (26).

In contrast to reports investigating the occurrence of autoimmune disorders in patients suffering from multiple sclerosis and treated with alemtuzumab, we observed no clinically apparent autoimmune thyroid disorders or idiopathic thrombocytopenic purpura in the present cohort (27). Possibly, the follow-up period of the present study was too short for these autoimmune events to occur. Another reason could be that patients with multiple sclerosis are susceptible to autoimmune disorders because of their genetic constitution.

The administration of rATG is associated with serious infusion-related side effects and the drug is relatively contra-indicated in patients with cardiac failure or fluid overload (11, 12). Infusion-related side effects in our study were less prevalent in patients treated with alemtuzumab compared with rATG therapy and subcutaneous administration of alemtuzumab therefore appears to be safe in frail patients and patients with cardiac morbidity. In this study, rATG was given as a bolus of 4 mg/kg. We cannot exclude the possibility that another dosing regimen such as a repeated, standard dose of rATG may have resulted in fewer infusion-related side effects (28).

We acknowledge the limitations of the current study. First, this was a retrospective single-center study. Second, several variables (including time period, the use of induction therapy and others) were different between the patients treated with alemtuzumab and the patients treated with rATG. A propensity score analysis was performed to correct for potential differences between the alemtuzumab and rATG group, but we cannot exclude the possibility that other (unmeasured) confounding factors influenced the outcomes of this analysis. Currently, these data offer the best available evidence for the treatment of AR with alemtuzumab as it is unlikely that a randomized controlled trial comparing alemtuzumab with other anti-rejection therapies will be performed anytime soon. Third, the allograft survival of patients who were treated with alemtuzumab seemed (although not significant) to be worse compared with rATG-treated patients. Again, we cannot exclude the possibility that inclusion of more patients may have resulted in a significant difference between the two groups. Fourth, in our study 93.8% of alemtuzumab-treated patients were treated with basiliximab induction therapy. In the United States, only 33.8% of kidney transplant recipients are treated with basiliximab, whereas 65.9% of patients receive induction therapy with T cell-depleting antibodies (29). We don't know the influence of this difference on the outcomes after alemtuzumab therapy for AR. Fifth, due the unavailability of data on DSAs in the rATG-treated patients, it was not possible to apply the Banff 2017 classification on biopsies of these patients which may have biased the diagnosis of ABMR.

To conclude, alemtuzumab therapy could be an alternative therapy to rATG for glucocorticoid-resistant or severe AR. This may be especially relevant for patients with a relative contraindication for rATG, including patients suffering from fluid overload or previous rATG treatment. Further studies, preferably multicenter randomized controlled trials, are necessary to explore the potential advantages of alemtuzumab for severe rejection.

## Data Availability Statement

The datasets generated for this study are available on request to the corresponding author.

## Ethics Statement

The studies involving human participants were reviewed and approved by Erasmus MC ethical board. Written informed consent for participation was not required for this study in accordance with the national legislation and the institutional requirements.

## Author Contributions

MZ participated in the research design, acquisition of the data, data analysis, and writing of the article. MC-V participated in the revision of the Banff classification of the kidney biopsies and critical revision of the manuscript. DR acquired the data of donor specific anti-HLA antibodies. KM and JR participated in data analysis and critical revision of the manuscript. MK, MH, MA, and CB participated in the critical revision of the manuscript. DH participated in the research design, data analysis, and critical revision of the manuscript. All authors contributed to the article and approved the submitted version.

### Conflict of Interest

DH has received grant support, lecture, and consulting fees from Astellas Pharma and Chiesi Pharmaceuticals, as well as a lecture fee from Hikma Pharma and grand support from Bristol Myers-Squibb. MC-V has received grant support from Astellas Pharma. MH has received grant support from Novartis and Shire and lecture fees from Astellas Pharma, Chiesi Pharmaceuticals, MSD, Sanofi/Genzyme, Shire, and Vifor Pharma. The remaining authors declare that the research was conducted in the absence of any commercial or financial relationships that could be construed as a potential conflict of interest.

## Supplementary Material

Figure S1 Survival plots of allograft- and patient survival in the period between 2002 and 2012 and 2012–2018. **(A)** Kaplan-Meier patient survival curve of patients treated with alemtuzumab for AR (2012–2018) and patients transplanted in the same period and not treated with alemtuzumab. From time point of acute rejection (alemtuzumab group) and time point of kidney transplantation (patients not treated with alemtuzumab). **(B)** Kaplan-Meier allograft survival curve (event = allograft loss, censored for death) of patients treated with alemtuzumab for AR (2012–2018) and patients transplanted in the same period and not treated with alemtuzumab. From time point of acute rejection (alemtuzumab group) and time point of kidney transplantation (patients not treated with alemtuzumab). **(C)** Kaplan-Meier patient survival curve of patients treated with rATG for AR (2002–2012) and patients transplanted in the same period and not treated with rATG. From time point of acute rejection (rATG group) and time point of kidney transplantation (patients not treated with rATG). **(D)** Kaplan-Meier allograft survival curve (event = allograft loss, censored for death) of patients treated with rATG for AR (2002–2012) and patients transplanted in the same period and not treated with rATG. From time point of acute rejection (rATG group) and time point of kidney transplantation (patients not treated with rATG).

Figure S2 Kaplan-Meier survival curves of patient survival of different age categories. **(A)** Patient survival of patients (<50 years at time of transplantation) treated with alemtuzumab for AR (2012–2018) and patients (<50 years at time of transplantation) transplanted in the same period and not treated with alemtuzumab. **(B)** Patient survival of patients (50–65 years at time of transplantation) treated with alemtuzumab for AR (2012–2018) and patients (50–65 years at time of transplantation) transplanted in the same period and not treated with alemtuzumab. **(C)** Patient survival of patients (>65 years at time of transplantation) treated with alemtuzumab for AR (2012–2018) and patients (>65 years at time of transplantation) transplanted in the same period and not treated with alemtuzumab.

Figure S3 The creatinine clearance (mL/min/1.73 m^2^) of patients treated with alemtuzumab **(A)** or rATG **(B)** for AR. The boxes represent median and IQR and the whiskers 5th and 95th percentile. N, number of patients with an eGFR; Baseline, best serum creatinine or eGFR in 3 months before AR; 0, serum creatinine or eGFR on day of AR; M3, 3 months after alemtuzumab or rATG (±4 weeks); M6, 6 months (±6 weeks) after alemtuzumab or rATG; M12, 12 months after alemtuzumab or rATG (±8 weeks). ^*^*p* <0.05, ^**^*p* = not significant.

Figure S4 Kaplan-Meier survival curve of allograft survival of alemtuzumab-treated patients with aTCMR or aABMR.

Figure S5 T- and B cells after alemtuzumab therapy. T- and B cells were measured every 3 months, until T cells were >200 × 10^6^/L. **(A)** Scatter dot plot of all measured T cells on different time points after alemtuzumab therapy. The horizontal line depicts the median. **(B)** Percent of patients with repopulation of T cells >200 × 10^6^/L in the year after alemtuzumab therapy. **(C)** Scatter dot plot of all measured B cells on different time points after alemtuzumab therapy. The horizontal line depicts the median. **(D)** Percent of patients with repopulation of B cells >100 × 10^6^/L in the year after alemtuzumab therapy.

Table S1 Patients with a second biopsy between methylprednisolone and alemtuzumab to confirm ongoing rejection.

Table S2 Cause of death after therapy with alemtuzumab or rATG.

Table S3 Univariable Cox proportional hazard regression analysis for risk of death within patients treated with alemtuzumab.

Table S4 Univariable Cox proportional hazard regression analysis for allograft loss in patients treated with alemtuzumab.

Table S5 Characteristics and statistical analysis of alemtuzumab-treated patients with HLA mismatch of 0–3, and patients with HLA mismatch of 4–6.

Table S6 Infections during the total follow-up after alemtuzumab and rATG treatment.

Table S7 Malignancies after alemtuzumab treatment.

Table S8 Malignancies after rATG treatment.

## Abbreviations

aABMR: Acute antibody-mediated rejection
aTCMR: Acute T cell-mediated rejection
AR: Acute kidney allograft rejection
C0: Pre-dose concentrations
CI: Confidence interval
CKD: Chronic kidney disease
CKD–EPI: Chronic kidney disease Epidemiology Collaboration
CMV: Cytomegalovirus
CsA: Cyclosporine A
DGF: Delayed graft function
DSA: Donor-specific anti-HLA antibodies
EBV: Epstein-Barr virus
eGFR: Estimated glomerular filtration rate
HR: Hazard ratio
IQR: Interquartile range
IVIg: Intravenous immunoglobulins
MMF: Mycophenolate mofetil
PRA: Panel reactive antibodies
PNF: Primary non-function
rATG: Rabbit anti-thymocyte globulin.
