# Supplementary material for: Comparison of Alemtuzumab and Anti-thymocyte Globulin Treatment for Acute Kidney Allograft Rejection
Source: Front Immunol. 2020 Jul 3;11:1332. doi: 10.3389/fimmu.2020.01332 (PMC7350932; doi:10.3389/fimmu.2020.01332)
Supplement: Figure S1 — Survival plots of allograft- and patient survival in the period between 2002 and 2012 and 2012–2018. (A) Kaplan-Meier patient survival curve of patients treated with alemtuzumab for AR (2012–2018) and patients transplanted in the same period and not treated with alemtuzumab. From time point of acute rejection (alemtuzumab group) and time point of kidney transplantation (patients not treated with alemtuzumab). (B) Kaplan-Meier allograft survival curve (event = allograft loss, censored for death) of patients treated with alemtuzumab for AR (2012–2018) and patients transplanted in the same period and not treated with alemtuzumab. From time point of acute rejection (alemtuzumab group) and time point of kidney transplantation (patients not treated with alemtuzumab). (C) Kaplan-Meier patient survival curve of patients treated with rATG for AR (2002–2012) and patients transplanted in the same period and not treated with rATG. From time point of acute rejection (rATG group) and time point of kidney transplantation (patients not treated with rATG). (D) Kaplan-Meier allograft survival curve (event = allograft loss, censored for death) of patients treated with rATG for AR (2002–2012) and patients transplanted in the same period and not treated with rATG. From time point of acute rejection (rATG group) and time point of kidney transplantation (patients not treated with rATG). [file Data_Sheet_2.PDF]

Supplemental digital content

Figures

SDC Figure 1

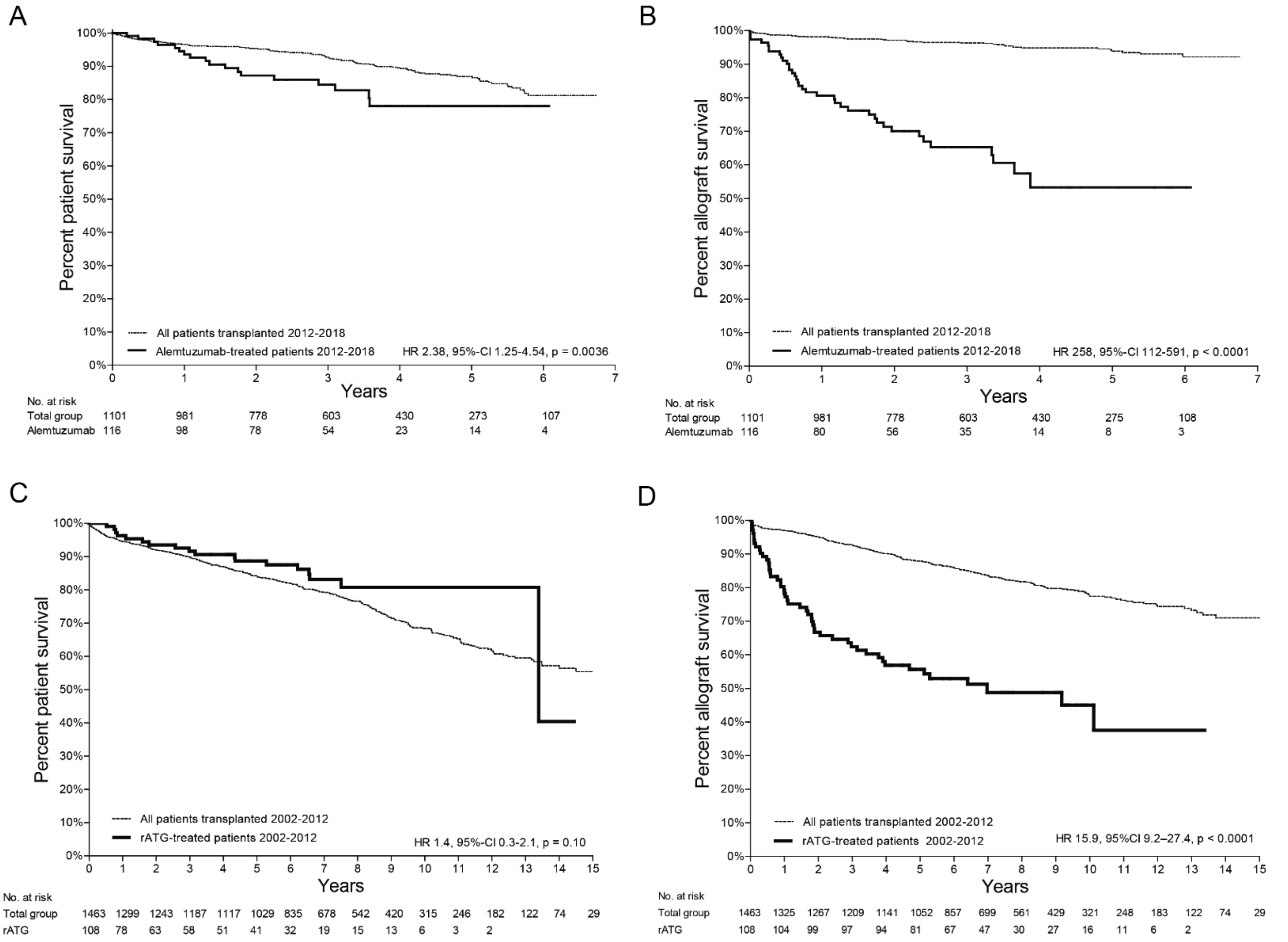
A
Percent patient survival
Years
All patients transplanted 2012-2018
Alemtuzumab-treated patients 2012-2018
HR 2.38, 95%-CI 1.25-4.54, p = 0.0036
No. at risk
Total group 1101 981 778 603 430 273 107
Alemtuzumab 116 98 78 54 23 14 4
B
Percent allograft survival
Years
All patients transplanted 2012-2018
Alemtuzumab-treated patients 2012-2018
HR 258, 95%-CI 112-591, p < 0.0001
No. at risk
Total group 1101 981 778 603 430 275 108
Alemtuzumab 116 80 56 35 14 8 3
C
Percent patient survival
Years
All patients transplanted 2002-2012
rATG-treated patients 2002-2012
HR 1.4, 95%-CI 0.3-2.1, p = 0.10
No. at risk
Total group 1463 1299 1243 1187 1117 1029 835 678 542 420 315 246 182 122 74 29
rATG 108 78 63 58 51 41 32 19 15 13 6 3 2
D
Percent allograft survival
Years
All patients transplanted 2002-2012
rATG-treated patients 2002-2012
HR 15.9, 95%CI 9.2–27.4, p < 0.0001
No. at risk
Total group 1463 1325 1267 1209 1141 1052 857 699 561 429 321 248 183 122 74 29
rATG 108 104 99 97 94 81 67 47 30 27 16 11 6 2

SDC Figure 2

A

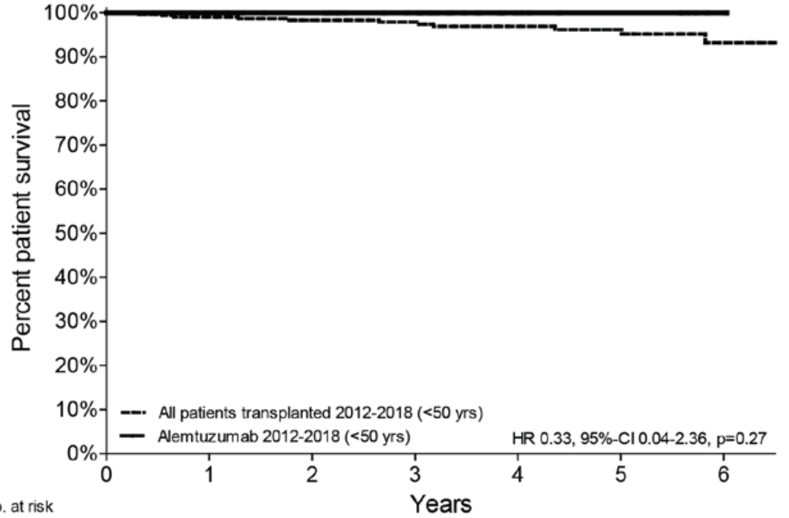

No. at risk							
Total group	329	304	249	203	149	98	39
Alemtuzumab	44	40	36	27	11	8	3

B

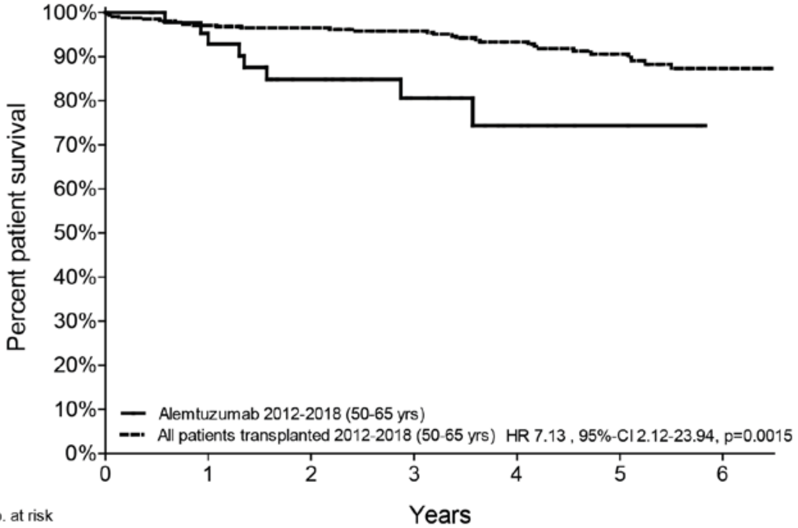

No. at risk							
Total group	413	374	312	256	191	129	60
Alemtuzumab	48	40	29	18	10	3	

C

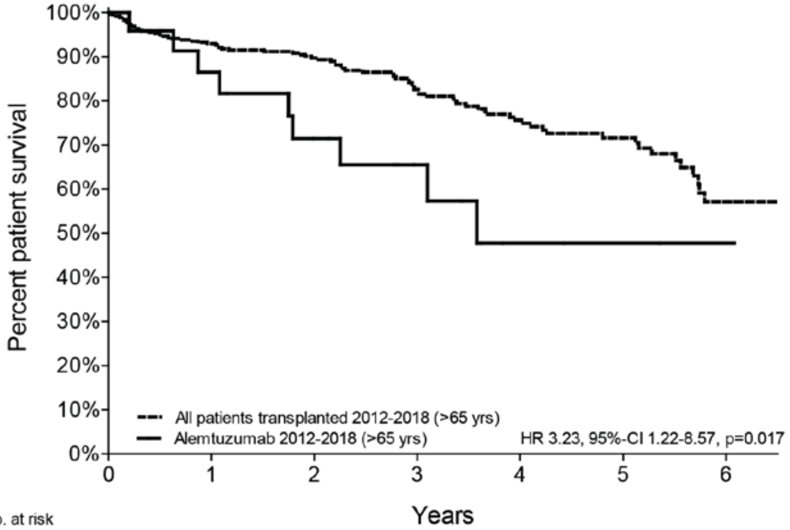

No. at risk							
Total group	359	322	240	163	109	68	23
Alemtuzumab	24	19	14	9	3	2	2

SDC Figure 3

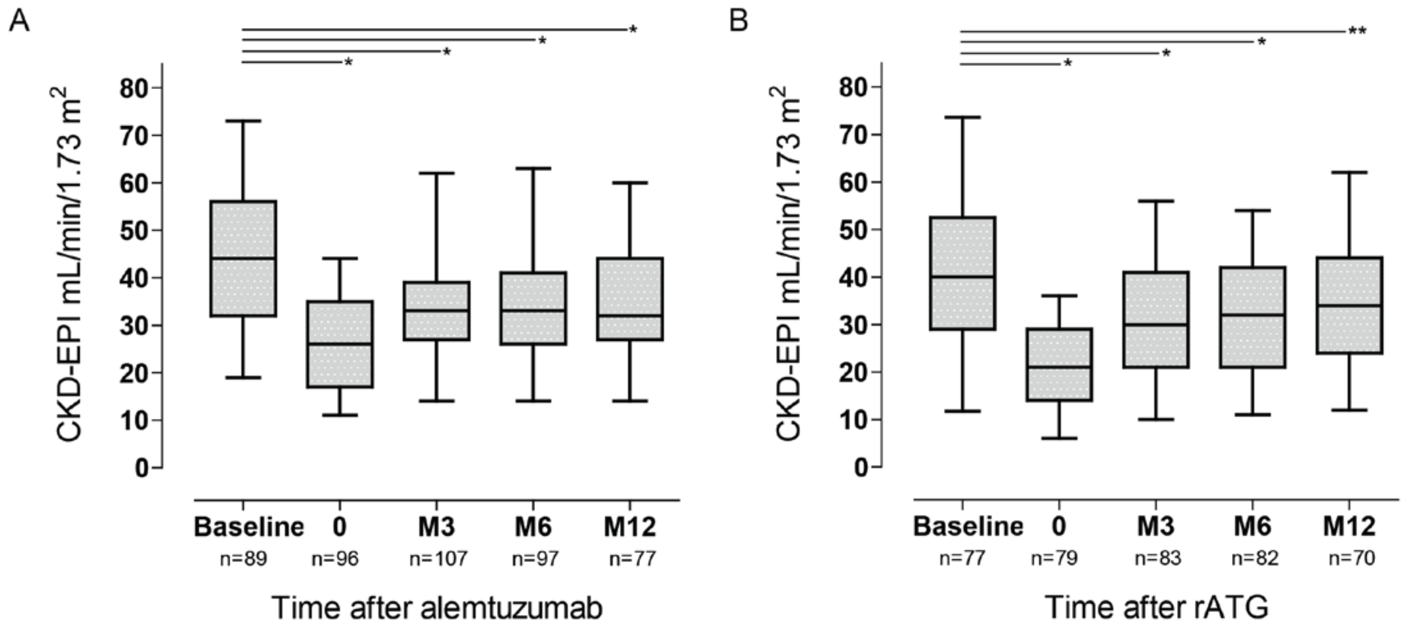

Methods

Of patients treated with alemtuzumab or rATG the allograft function (estimated glomerular filtration rate (eGFR; Chronic Kidney Disease Epidemiology Collaboration [CKD-EPI][1]) was assessed. Baseline eGFR was defined as the highest eGFR in the three months prior to AR. Delayed graft function (DGF) was defined as need for dialysis in the first week after transplantation. A linear mixed-effect model with patient specific random intercepts was constructed to analyze eGFR over time. Gender, ethnicity, primary kidney disease, age and donor type were included as covariates in the mixed effect model.

Results

Kidney function one year after alemtuzumab treatment was significantly worse compared to baseline kidney function. A good response (i.e. a return to >75% of baseline eGFR one year after alemtuzumab treatment) was seen in 46% of alemtuzumab-treated patients. Twenty-three percent of the patients had a partial response (return to 50-75% of baseline eGFR) after alemtuzumab, and 31% of the patients showed no response (return to baseline <50% of eGFR). Thirty-three patients (28.4%) had DGF during AR. Eight of these 33 patients lost their allograft in the first year, including six patients with PNF. Serum creatinine of the remaining patients with DGF one year after alemtuzumab was 147 µmol/L (IQR 141-165).

References

1. Levey AS, Stevens LA, Schmid CH, et al. A new equation to estimate glomerular filtration rate. Ann Intern Med. 2009;150(9):604-612.

SDC Figure 4

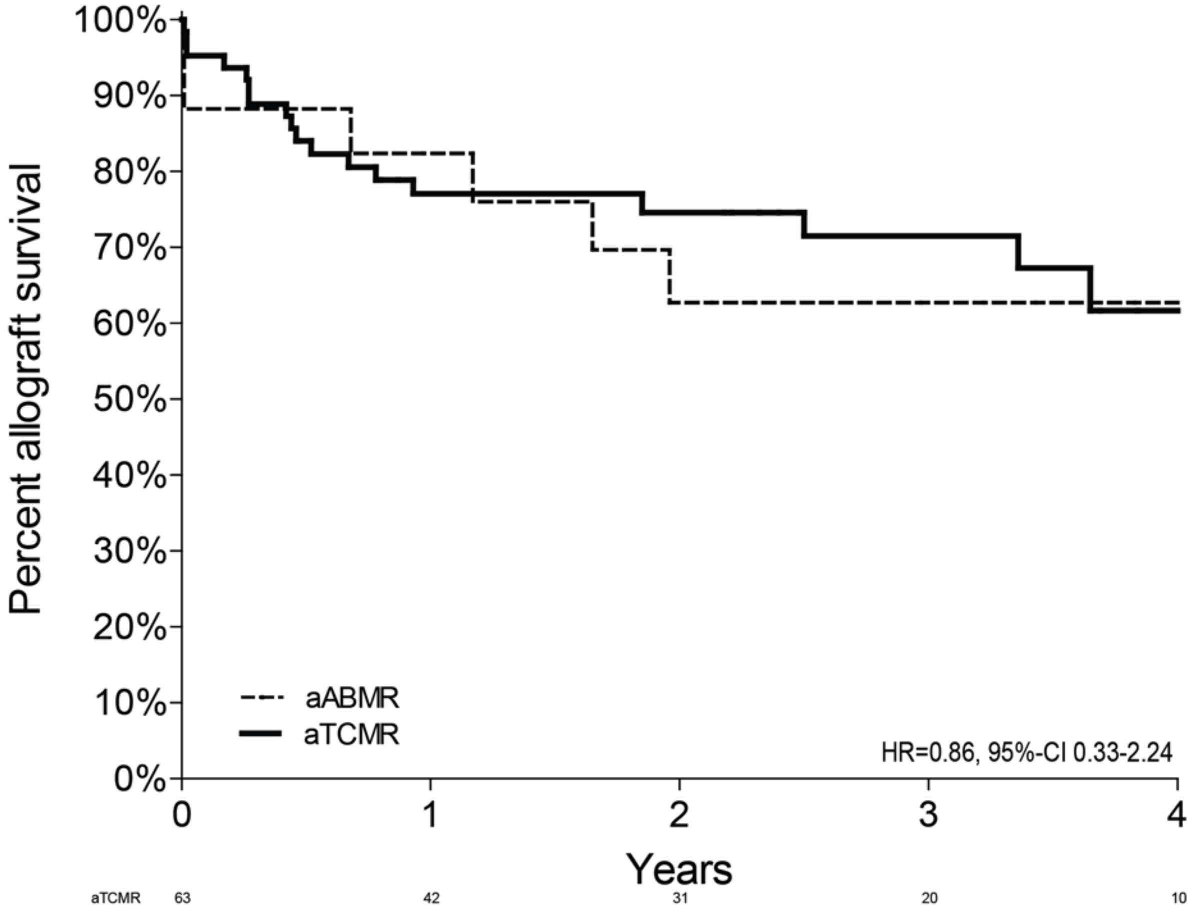

aTCMR	63	42	31	20	10
aABMR	18	14	10	7	3

SDC Figure 5

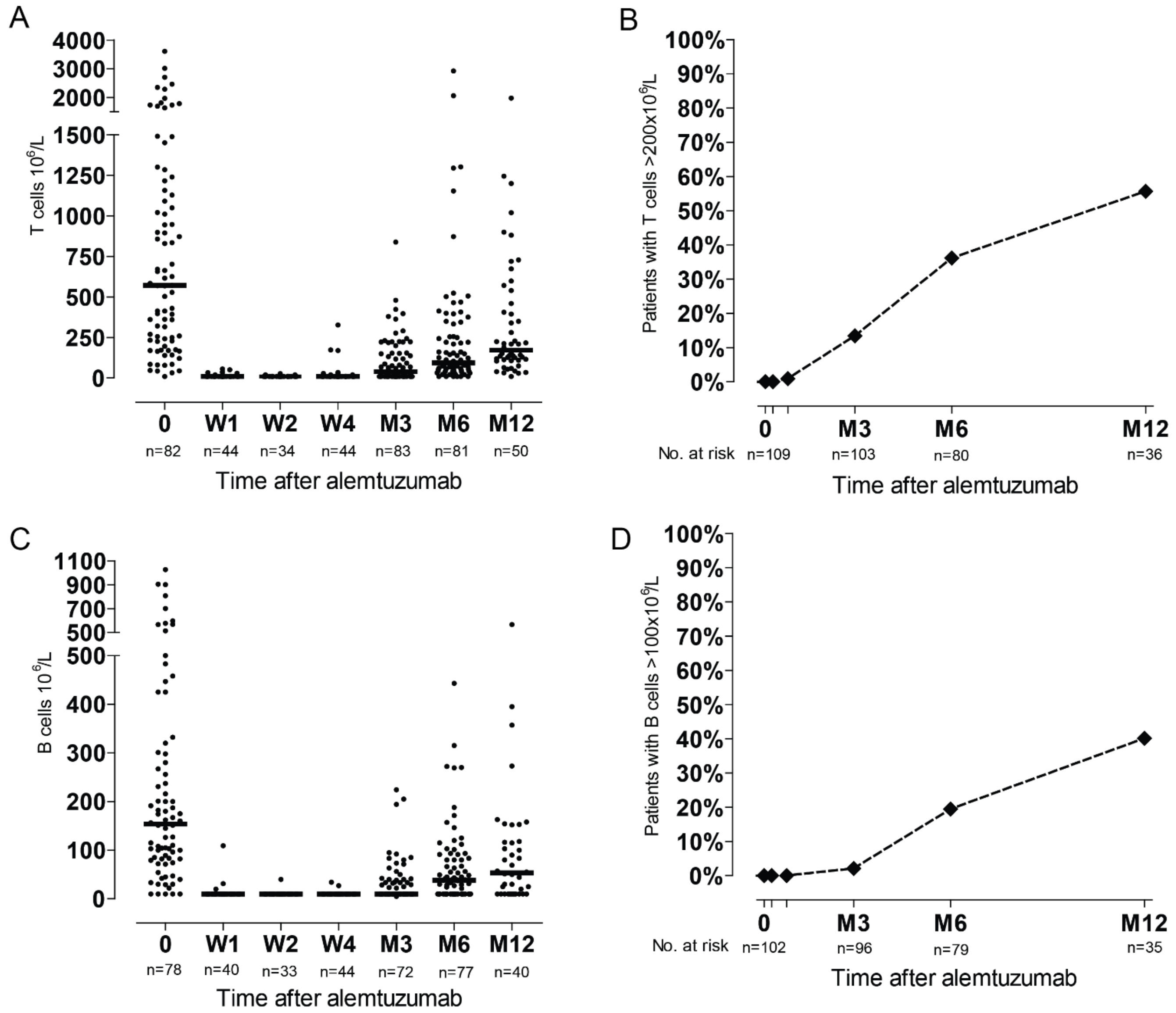
A
4000
3000
2000
1500
1250
1000
750
500
250
0
T cells 10^6/L
0 W1 W2 W4 M3 M6 M12
n=82 n=44 n=34 n=44 n=83 n=81 n=50
Time after alemtuzumab
B
100%
90%
80%
70%
60%
50%
40%
30%
20%
10%
0%
Patients with T cells >200x10^6/L
0 M3 M6 M12
No. at risk n=109 n=103 n=80 n=36
Time after alemtuzumab
C
1100
900
700
500
500
400
300
200
100
0
B cells 10^6/L
0 W1 W2 W4 M3 M6 M12
n=78 n=40 n=33 n=44 n=72 n=77 n=40
Time after alemtuzumab
D
100%
90%
80%
70%
60%
50%
40%
30%
20%
10%
0%
Patients with B cells >100x10^6/L
0 M3 M6 M12
No. at risk n=102 n=96 n=79 n=35
Time after alemtuzumab
