# Supplementary material for: Comparison of Alemtuzumab and Anti-thymocyte Globulin Treatment for Acute Kidney Allograft Rejection
Source: Front Immunol. 2020 Jul 3;11:1332. doi: 10.3389/fimmu.2020.01332 (PMC7350932; doi:10.3389/fimmu.2020.01332)
Supplement: Table S1 — Patients with a second biopsy between methylprednisolone and alemtuzumab to confirm ongoing rejection. [file Data_Sheet_1.pdf]

Supplemental digital content

Tables

SDC Table 1. Patients with a second biopsy between methylprednisolone and alemtuzumab to confirm ongoing rejection.

Patient no.	1st Banff diagnosis	Treatment	Days between 1st and 2nd biopsy	2nd Banff diagnosis
1	aABMR	Methylprednisolon 3 x 1000 mg	8	aTCMRIIA
2	aABMR	Methylprednisolon 3 x 1000 mg + IVIg	50	aABMR
3	aABMR	Methylprednisolon 6 x 1000 mg	7	aTCMRIIB
4	aTCMRIB	Methylprednisolon 3 x 1000 mg	85	aTCMRIB
5	aTCMRIIA	Methylprednisolon 6 x 1000 + IVIg	16	aTCMRIIA
6	aABMR	Methylprednisolon 3 x 1000 mg + IVIg	61	aABMR
7	aTCMRIIB	Methylprednisolon 3 x 1000 mg + IVIg	111	chronic active TCMR
8	aTCMR (biopsy missing)	Methylprednisolon 3 x 1000 mg	77	aTCMRIB
9	aTCMRIA	Methylprednisolon 3 x 1000 mg + IVIg	52	c/aABMR, borderline TCMR
10	aTCMRIB	Methylprednisolon 3 x 1000 mg	80	aTCMRIIA, aABMR
11	aTCMRIB	Methylprednisolon 6 x 1000 mg	17	borderline TCMR
12	aTCMRIB	Methylprednisolon 3 x 1000 mg	54	aTCMRIB
13	borderline aTCMR	Methylprednisolon 3 x 1000 mg	86	Acute tubular necrosis
14	aABMR	Methylprednisolon 3 x 1000 mg + IVIg	33	aABMR
15	aTCMRIA	Methylprednisolon 3 x 1000 mg	126	chronic active TCMR
16	aTCMRIIA	Methylprednisolon 3 x 1000 mg	9	aTCMRIIA
17	aTCMRIB	Methylprednisolon 3 x 1000 mg	111	chronic active TCMR
18	aTCMRIIB	Methylprednisolon 3 x 1000 mg + IVIg	7	ABMR (biopsy missing)

aABMR active antibody mediated rejection; aTCMR acute T cell mediated rejection; IVIg intravenous immunoglobulins

SDC Table 2. Cause of death after therapy with alemtuzumab or rATG

Adverse events	Alemtuzumab	rATG
Patient death - no (%)	18 (15.5)	17 (16.5)
Time after therapy - yr.	1.45 (0.92-2.93)	3.1 (1-6.3)
Cause of death - no.		
Infectious	7	5
Carcinoma	4	2
Cardiovascular	2	3
Hepatic failure	1	1
Allograft failure	2	0
Unknown	2	6

Data are numbers (percentage) and median (interquartile range).

SDC Table 3. Univariable Cox proportional hazard regression analysis for risk of death within patients treated with alemtuzumab

**Variable (reference category)**	**Exp (B)**	**95% CI for Exp (B)**	**p-value**
Patient characteristics			
Recipient age at transplantation (per yr)	1.09	1.04-1.14	<0.0001
Recipient age at acute rejection (per yr)	1.09	1.04-1.14	<0.0001
Donor age (per yr)	1.00	0.97-1.03	1.00
Gender (female)	0.44	0.29-1.73	0.44
Ethnicity (Caucasian)	0.40	0.12-1.37	0.14
Transplant number (1)	0.55	0.16-1.88	0.34
PRA current (<6%)	1.07	0.36-3.23	0.90
Transplant characteristics			
Type donor (living)	1.20	0.46-3.16	0.71
HLA mismatch (per HLA mismatch)	0.88	0.64-1.19	0.40
Therapy characteristics			
Maintenance therapy (TAC+MMF±glucocorticoids)	5.70	0.76-42.83	0.09
Glucocorticoid maintenance (no)	1.63	0.47-5.60	0.44
Rejection characteristics			
Timing rejection (< 3 months)	0.52	0.20-1.32	0.17
Type rejection			0.41
DSA vs no DSA	0.40	0.11-1.44	0.16
CKD at time rejection (CKD 3)			0.30
Δ eGFR Baseline- moment of rejection			0.99
Interval methylprednisolon-alemtuzumab	1.00	0.99-1.02	0.83
Allograft loss	1.18	0.47-2.94	0.72
T cells after 3 months***	1.00	0.99-1.01	0.85

Increasing age at time of transplantation or AR resulted in increased risk of death. Ethnicity is caucasian or non-caucasian. Transplant number is 1 or >1. PRA current is < 6% or ≥6%. Type donor is living or deceased. Maintenance therapy is TAC+MMF± glucocorticoids or the other combinations of drugs. Glucocorticoid maintenance is use at the time of rejection. Timing of the rejection is <3 or > 3months after transplantation. Type rejection is aTCMR, ABMR, or mixed. CDK at time rejection is CKD1+2+3, CKD4, CKD5, and delayed graft function+ primary non-function. Interval between methylprednisolon and alemtuzumab is days. T cells three months after alemtuzumab is a continue variable. Data of rATG-treated patients were described previously[10]. CKD chronic kidney disease; MMF mycophenolate mofetil; PRA panel reactive antigen; TAC tacrolimus

SDC Table 4. Univariable Cox proportional hazard regression analysis for allograft loss in patients treated with alemtuzumab

Variable (reference category)	Exp (B)	95%-CI for Exp (B)	p-value
**Patient characteristics**			
Recipient age at transplantation (per yr)	0.97	0.95-0.99	0.002
Recipient age at acute rejection (per yr)	0.97	0.95-0.99	0.003
Donor age (per yr)	0.99	0.97-1.01	0.27
Gender (female)	0.99	0.53-1.86	0.97
Ethnicity (Caucasian)	1.11	0.57-2.15	0.77
Transplant number (1)	1.1	0.53-2.23	0.81
PRA actual (<6%)	1.35	0.64-2.87	0.43
**Transplant characteristics**			
Type donor (living)	1.48	0.77-2.85	0.24
HLA mismatch (per HLA mismatch)	0.72	0.58-0.89	0.002
HLA mismatch (0-3)	0.34	0.17-0.65	0.001
**Therapy characteristics**			
Maintenance therapy (TAC+MMF± glucocorticoids)	1.19	0.55-2.60	0.66
Glucocorticoid maintenance (no)	0.32	0.17-0.60	<0.0001
Frequency alemtuzumab (1)	1.19	0.56-2.54	0.65
**Rejection characteristics**			
Timing rejection (< 3 months)	1.39	1.01-1.91	0.04
Type rejection (aTCMR)			0.19
DSA vs no DSA	1.36	0.66-2.81	0.40
CKD at time rejection (CKD1-3)			0.20
Δ eGFR baseline-moment of rejection			0.004
25 till 50% drop versus <25% drop	0.91	0.33-2.53	0.81
>50% drop versus <25% drop	3.36	1.29-8.75	0.008
Interval methylprednisolon-alemtuzumab	1.00	0.99-1.01	0.49

Transplant number is 1 or >1. PRA current is < 6% or ≥6%. Type donor is living or deceased. Maintenance therapy is TAC+MMF± glucocorticoids or the other combinations of drugs. Glucocorticoid maintenance is use at the time of rejection. Frequency of alemtuzumab is 1 or >1. Timing of the rejection is <3 or > 3 months after transplantation. Type rejection is aTCMR, ABMR, or mixed. CDK at time rejection is CKD1+2+3, CKD4, CKD5+ delayed graft function+ primary non-function. Interval between methylprednisolon and alemtuzumab is days. Data of rATG-treated patients were described previously[10]. CKD chronic kidney disease; DSA de novo donor specific antibodies; MMF mycophenolate mofetil; PRA panel reactive antibodies; TAC tacrolimus

SDC Table 5. Characteristics and statistical analysis of alemtuzumab-treated patients with HLA mismatch of 0-3, and patients with HLA mismatch of 4-6

Characteristic	HLA MM 0-3 (n=57)	HLA MM 4-6 (n=58)	p-value
Recipient age at transplantation- yr.	54 (38-63)	57 (40-63)	0.31
Recipient age at rejection- yr.	54 (38-63)	58 (42-64)	0.28
Donor age - yr.	54 (40-63)	54 (40-63)	0.59
Female sex - no. (%)	25 (43)	22 (38)	0.71
Cause of ESRD - no.			
DM/HTN/GN/PKD/reflux/other/unknown	12/9/12/4/5/16/0	14/13/9/5/2/11/4	0.28
Ethnic distribution - no.			
Caucasian/Black/Asian/Arab/other	40/8/4/6/0	39/9/4/5/1	1.00
Transplant number - no.			
1/2/3	41/12/5	47/10/1	0.22
Preemptive kidney transplantation - no. (%)	22 (38)	19 (32.8)	0.70
Donor type - no.			
LR/LUR/DBD/DCD	24/13/6/15	3/42/6/7	<0.001
Living/deceased	37/21	45/13	0.15
HLA mismatch - no.			
HLA A: 0/1/2	22/33/3	4/28/26	<0.001
HLA B: 0/1/2	10/43/5	0/10/48	<0.001
HLA DR: 0/1/2	19/36/3	2/19/37	<0.001
PRA actual - no. (%)			0.35
0-5%	44 (75.9)	49 (84.4)	
6-83%	13 (22.4)	9 (15.5)	
84-100%	1 (1.7)	0 (0)	
PRA peak - no. (%)			0.29
0-5%	33 (56.9)	36 (62.1)	
6-83%	14 (24.1)	17 (29.3)	
84-100%	11 (19.0)	5 (8.6)	
De novo DSA (ABMR and mixed type rejections)			
DSA+/non-donor HLA antibodies/no DSA/not tested	6/4/2/2	14/1/3/3	0.24
Class I/II/I+II	1/3/2	3/9/2	0.62
CMV IgG serostatus recipient - no. (%)			
Positive	43 (74)	41 (71.9)	0.84

EBV IgG serostatus recipient - no. (%)			
Positive	52 (91.2)	55 (96.5)	0.44
Maintenance therapy (TAC+MMF± glucocorticoids) - no. (%)	49 (84.4)	47 (79.3)	0.63
Glucocorticoid maintenance - no. (%)	44 (75.9)	49 (84.5)	0.35
Timing rejection (< 3 months) - no. (%)	31 (53.4)	32 (55.2)	1.00
Type rejection	34/6/8	32/10/11	0.54
CKD at time rejection	17/21/20	20/18/19	0.80
Interval methylprednisolon-alemtuzumab	6 (4-24)	7 (4-22)	0.80

Data are numbers (%) or median (interquartile range). Type rejection is aTCMR, ABMR, or mixed. CDK at time rejection is CKD1+2+3, CKD4, CKD5+ delayed graft function+ primary non-function. Interval between methylprednisolon and alemtuzumab is days. CMV cytomegalovirus; DBD donation after brain death; DCD donation after circulatory death; DM diabetes mellitus; DSA de novo donor specific antibodies; EBV Epstein-Barr virus; ESRD end stage renal disease; GN glomerulonephritis; HLA human leucocyte antigen; HTN hypertensive nephropathy; LR living related; LUR living unrelated; MMF mycophenolate mofetil; PKD polycystic kidney disease; PRA panel reactive antibody; rATG rabbit anti-thymocyte globulin; TAC tacrolimus

SDC Table 6. Infections during the total follow-up after alemtuzumab and rATG treatment

Infections	Alemtuzumab	rATG
Infection in the first year - no.	96	124
Viral	14	19
Fungal	3	8
Bacterial	79	97
Blood	3	8
Urinary tract/urosepsis	47	51
Skin and soft tissue	5	9
Lung	16	15
Tuberculosis	2	0
Other/unknown	6	14
Infection in the first 15 days	9	29
CMV infections		
CMV reactivation - no. (%)	25 (21.6)	27 (25)
CMV disease - no. (%)	2 (1.7)	0
Primary CMV infection - no. (%)	2 (1.7)	2 (1.9)
CMV, time after therapy	76 (52-167)	32 (19-74)
EBV infections - no. (%)	0	1 (9)
BK infections		
BK viremia - no. (%)	20 (17.2)	6 (5.6)
BK viremia, time after therapy	106 (59-186)	458 (322-844)

Data are numbers (%) or median (interquartile range). CMV, EBV and BK virus infections were scored apart from the infections during the total follow-up, in the first year, and after the first year.

SDC Table 7. Malignancies after alemtuzumab treatment

Malignancy	Months after alemtuzumab	Months after transplantation	Age of patient
Lung cancer	9	10	57
Lung cancer	27	38	61
Pancreatic cancer	37	43	73
Breast cancer	28	32	65
Prostate carcinoma	43	44	80
Adenocarcinoma of unknown primary*	38	46	60
Colon cancer**	8	58	76

Age of patient is at the time of malignancy. *No biopsy of the metastasis was taken. **This patient had pre-transplantation colon cancer and developed metastasis of colon cancer after alemtuzumab.

SDC Table 8. Malignancies after rATG treatment

Malignancy	Months after rATG	Months after transplantation	Age of patient
Endometrial carcinoma	67	69	57
Adenocarcinoma lung	68	157	61
Non seminoma testis	17	23	44
Colon carcinoma	21	21	45
Rectal carcinoma	17	62	52
Meningioma	28	44	62
Renal carcinoma	107	107	34
Renal carcinoma	77	77	69
Renal carcinoma	140	140	55
Renal carcinoma	144	144	56
Prostatic carcinoma	10	35	54
Prostatic carcinoma	77	77	69
Non-Hodgkin lymphoma	78	79	56
EBV related B-lymphoma	14	14	65

Age of patient is at the time of malignancy. The Epstein-Barr virus (EBV)-related lymphoma was in an IgG seropositive patient and occurred fourteen months after treatment with rATG and was treated with irradiation.
